# DMM Outstanding Paper Prize 2020 winner: Sarah Colijn

**DOI:** 10.1242/dmm.049024

**Published:** 2021-04-15

**Authors:** Rachel Hackett

**Affiliations:** The Company of Biologists, Bidder Building, Station Road, Cambridge CB24 9LF, UK

## Abstract

Disease Models & Mechanisms (DMM) is delighted to announce that the winner of the DMM Prize 2020 is Sarah Colijn, for her paper entitled ‘Cell-specific and athero-protective roles for RIPK3 in a murine model of atherosclerosis’ (Colijn et al., 2020b). The prize of $1000 is awarded to the first author of the paper that is judged by the journal's editors to be the most outstanding contribution to the journal that year. To be considered for the prize, the first author must be a student or a postdoc of no more than 5 years standing.

**Figure:**
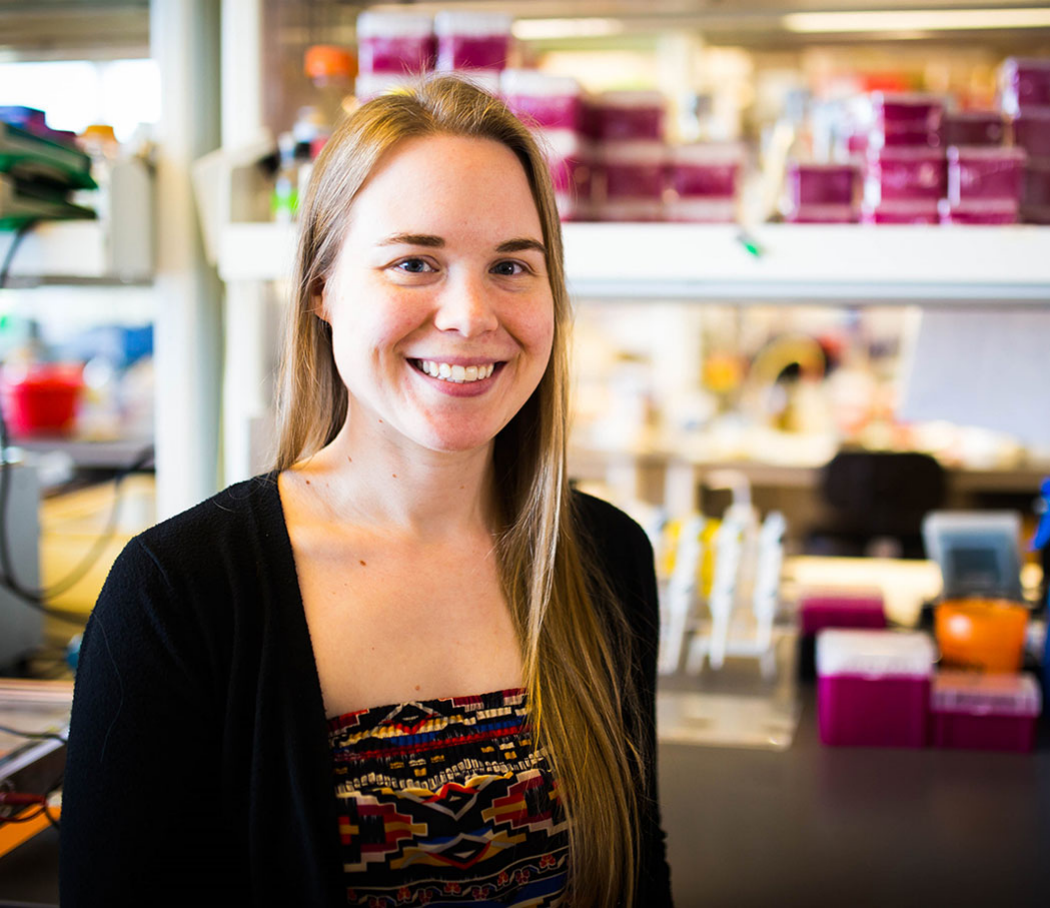
**Sarah Colijn**

## Outstanding contribution

By the time Sarah Colijn was twelve years old, she had already lived with her family in five different states. At age twelve, Sarah and her family finally settled down in Norman, Oklahoma – the land of Sooner football and tornadoes. Sarah was always greatly attracted to the idea of a career in a STEM field, but it wasn't until she read an article about malaria in an edition of *National Geographic* that she fell in love with the idea of biomedical research.

**Box 1. DMM Prize 2020 shortlist**

**Winner:**

**Cell-specific and athero-protective roles for RIPK3 in a murine model of atherosclerosis.**

Sarah Colijn, Vijay Muthukumar, Jun Xie, Siqi Gao, Courtney T. GriffinDisease Models & Mechanisms 2020 13: dmm041962 doi:10.1242/dmm.041962

**Also shortlisted by our Editor team**:

**Misfolded α-synuclein causes hyperactive respiration without functional deficit in live neuroblastoma cells**

Cathryn L. Ugalde, Sarah J. Annesley, Shane E. Gordon, Katelyn Mroczek, Matthew A. Perugini, Victoria A. Lawson, Paul R. Fisher, David I. Finkelstein, Andrew F. HillDisease Models & Mechanisms 2020 13: dmm040899 doi:10.1242/dmm.040899

**A comprehensive study of phospholipid fatty acid rearrangements in metabolic syndrome: correlations with organ dysfunction**

Amélie Bacle, Linette Kadri, Spiro Khoury, Romain Ferru-Clément, Jean-François Faivre, Christian Cognard, Jocelyn Bescond, Amandine Krzesiak, Hugo Contzler, Nathalie Delpech, Jenny Colas, Clarisse Vandebrouck, Stéphane Sébille, Thierry FerreiraDisease Models & Mechanisms 2020 13: dmm043927 doi:10.1242/dmm.043927

**Identification of *MYOM2* as a candidate gene in hypertrophic cardiomyopathy and Tetralogy of Fallot, and its functional evaluation in the *Drosophila* heart**

Emilie Auxerre-Plantié, Tanja Nielsen, Marcel Grunert, Olga Olejniczak, Andreas Perrot, Cemil Özcelik, Dennis Harries, Faramarz Matinmehr, Cristobal Dos Remedios, Christian Mühlfeld, Theresia Kraft, Rolf Bodmer, Georg Vogler, Silke R. SperlingDisease Models & Mechanisms 2020 13: dmm045377 doi:10.1242/dmm.045377

**Integrated lipidomic and transcriptomic analyses identify altered nerve triglycerides in mouse models of prediabetes and type 2 diabetes**

Phillipe D. O'Brien, Kai Guo, Stephanie A. Eid, Amy E. Rumora, Lucy M. Hinder, John M. Hayes, Faye E. Mendelson, Junguk Hur, Eva L. FeldmanDisease Models & Mechanisms 2020 13: dmm042101 doi:10.1242/dmm.042101

**Deficiency in the endocytic adaptor proteins PHETA1/2 impairs renal and craniofacial development**

Kristin M. Ates, Tong Wang, Trevor Moreland, Rajalakshmi Veeranan-Karmegam, Manxiu Ma, Chelsi Jeter, Priya Anand, Wolfgang Wenzel, Hyung-Goo Kim, Lynne A. Wolfe, Joshi Stephen, David R. Adams, Thomas Markello, Cynthia J. Tifft, Robert Settlage, William A. Gahl, Graydon B. Gonsalvez, May Christine Malicdan, Heather Flanagan-Steet, Y. Albert PanDisease Models & Mechanisms 2020 13: dmm041913 doi:10.1242/dmm.041913

**The zebrafish as a novel model for the *in vivo* study of *Toxoplasma gondii* replication and interaction with macrophages**

Nagisa Yoshida, Marie-Charlotte Domart, Christopher J. Peddie, Artur Yakimovich, Maria J. Mazon-Moya, Thomas A. Hawkins, Lucy Collinson, Jason Mercer, Eva-Maria Frickel, Serge MostowyDisease Models & Mechanisms 2020 13: dmm043091 doi:10.1242/dmm.043091

Sarah attended the University of Oklahoma with a National Merit Scholarship. In 2013, she received her bachelor's degree in Biochemistry with a minor in Physics. She then started graduate school at the University of Oklahoma Health Sciences Center and joined the lab of Dr Courtney Griffin at the Oklahoma Medical Research Foundation, where she discovered that her scientific passion is studying the blood vasculature.

Sarah's first project involved exploring the role of chromatin-remodeling complexes in vascular development and vessel stability. By using genetically-modified mouse embryos as her animal model, Sarah found that the executioner protein RIPK3 – from the cell death pathway known as necroptosis – is carefully regulated by chromatin-remodelling complexes at mid-gestation to prevent vascular haemorrhage. Furthermore, she and her colleagues discovered that endothelial RIPK3 levels are particularly sensitive to hypoxia, i.e. physiologically low levels of blood oxygen, as observed during gestation. This bolstered the finding that endothelial RIPK3 levels must be suppressed at mid-gestation to support embryonic survival and vascular integrity (Colijn et al., 2020a).

Sarah's second project was to investigate the detrimental role of RIPK3 in a murine model of atherosclerosis. Necroptosis – and, thus, by association RIPK3 – is considered to be harmful because it can exacerbate inflammation and, theoretically, make atherosclerosis worse. It has been proposed that necroptosis and RIPK3 are viable targets for drugs that prevent atherosclerosis as macrophages are able to undergo necroptosis within atherosclerotic plaques. However, to Sarah's great surprise, she found that inhibition of RIPK3 in certain cell types has unexpected and pro-atherogenic effects, indicating that it is a poor target for drug design. In particular, deletion of RIPK3 in either macrophages or endothelial cells revealed that RIPK3 has an athero-protective role in these cell types (Colijn et al., 2020b). Sarah's work challenges research regarding atherosclerosis and necroptosis to rethink the role of RIPK3 and to acknowledge that it can function in a non-pathological way; she and her colleagues suspect that RIPK3 functions in a multitude of pathways that have very little to do with necroptosis. In fact, this is becoming more and more clear as Dr Griffin's group continues to study RIPK3 in the vasculature during embryogenesis and postnatally. Sarah is honoured that her work with RIPK3 and atherosclerosis was chosen as the winner of the 2020 DMM Prize as the most outstanding contribution to the journal.

After receiving her Ph.D. in 2019, Sarah began her postdoctoral position at Washington University in St. Louis with Dr Amber Stratman. She has shifted animal models and now works with zebrafish to uncover blood-flow-dependent mechanisms that regulate vascular development. Sarah hopes to carry on the legacy of outstanding female mentorship she has received from Drs Griffin and Stratman as she continues in her own academic career.
